# Health of Cities

**Published:** 1877-07

**Authors:** 


Health of Cities.—The public health of the foregoing cities was not in such a satisfactory condition during March as it was in February, if we may judge by the death-rates; the inclement weather of March was probably one important factor in producing an incjeased mortality.

In New York, the deaths were mostly caused by phthisis, pneumonia, and bronchitis; diptheria and croup were more fatal than in February; scarlatina has steadily diminished since January.

In Philadelphia, the chief causes of death were phthisis, pneumonia, bronchitis, diptheria, small-pox, croup, typhoid fever, scarlatina,—all of them more fatal than in February.

In Brooklyn, the mortality from the seven chief zymotic diseases, was nearly the same as in February. Diptheria and croup have increased considerably. Scarlitina is less. The relative order of prevalent diseases is as follows: phthisis, diptheria (and croup), pneumonia, scarlatina, bronchitis.

In Chicago, scarlatina heads the list but is declining.

In Boston, scarlatina remains very near the low place in the list which it reached in February.

In Providence, the death-rate for the month was only 16.3 per 1000 of Population. The mortality was chiefly from diseases of the respiratory system. Diptheria was also more fatal than in February.

In Massachusetts cities other than Boston, phthisis, diptheria (and croup), and pneumonia were the chief causes of death. Scarlstina has subsided.—*Boston Med. Journal.*

